# Going South in Winter

**Published:** 1878-01

**Authors:** 


Multitudes leave our northern regions, and proceed southward in the early winter, to escape the rigors of our climate. The change may be beneficial, in fact it often is, particularly with patients to whom warmth is life and vigor and strength, and whom the cold pinches and makes wretched. Florida is the attractive spot now-a-days, and by the aid of the Old Dominion Steamship Company, all parts of the State are reached with small annoyance or fatigue. No safer or better equipped steamers leave our harbor than those of this company.

Among the pleasant features of the trip by this line may be mentioned the passing by daylight in full view of Long Branch, the beautiful Highlands of Neversink, the beacons of the Jersey and Virginia coasts, and Fortress Monroe, the center of active operations in Hampton Roads during the late civil war, where the naval battle between the "Monitor" and "Merrimac" took place. The town of Norfolk, some two hundred years old, and the second largest cotton port in the United States, is an interesting spot, as is also the city of Portsmouth, where connection is made with the system of railroads through the seaboard States.

From here the route lies through old settled portions of Virginia and North Carolina, passing through the towns of Suffolk, Weldon, Goldsboro and Wilmington. The latter city is the great depot for naval stores, and has a large export trade with England and the continent in these products. The traveller next passes through a pleasant part of the State of South Carolina to the City of Charleston, visiting, if so inclined, the ruins of Forts Sumter and Moultrie, and many other points of interest. Savannah, in the State of Georgia, one of the most beautiful cities in the South, is next reached. The cities of Augusta and Macon may also be visited to advantage, the delightful climate rendering them particularly agreeable to invalids. Aiken, a noted retreat for invalids, is within a few miles of Augusta. After leaving Savannah, Florida—the fitting end of the journey—is reached. The many fine hotels in the City of Jacksonville, and its accessible position, being at the gateway of the State, as it were, makes it usually the base for further excursions. A number of boats perform regular daily service to all the landings on the St. John and Ocklawaha Rivers. The traveller may, after leaving Wilmington, continue to Columbia, the capital of S. C., and thence to Augusta and Savannah, or he may take the regular steamers from Charleston or Savannah to Jacksonville. From Savannah the route is through the inland waters near the coast, amid the tropical scenery peculiar to the region. The route from Charleston is by sea, but in sight of the coast for the entire distance.

Both routes are delightful, and the cost is moderate. We prefer the sea-voyage from Charleston, because of the tonic power of the sea air. The accommodations are excellent all along the route. Invalids and others visiting the south will do well to heed these suggestions.

